# Coevolution of the Asymmetric Morphology and the Behaviour of Simple Predator Agents in Predator-Prey Pursuit Problem

**DOI:** 10.1155/2019/1538757

**Published:** 2019-05-06

**Authors:** Milen Georgiev, Ivan Tanev, Katsunori Shimohara

**Affiliations:** Graduate School of Science and Engineering, Doshisha University, 1-3 Tatara-Miyakodani, Kyotanabe, Kyoto 610-0321, Japan

## Abstract

Humanity has long strived to create microscopic machines for various purposes. Most prominent of them employ nanorobots for medical purposes and procedures, otherwise deemed hard or impossible to perform. However, the main advantage of this kind of machines is also their main drawback—their small size. The miniature scale, they work in, brings many problems, such as not having enough space for the computational power needed for their operation or the specifics of the laws of physics that govern their behaviour. In our study, we focus on the former challenge, by introducing a new standpoint to the well-studied predator-prey pursuit problem using an implementation of very simple predator agents. Intended to model the small-scale (micro and nano) robots, these agents are morphologically simple—they feature a single line-of-sight sensor. The behaviour of the predator agents is simple as well—the (few) perceived environmental variables are mapped directly into corresponding pairs of rotational velocities of the wheels' motors. We implemented genetic algorithm to evolve such a mapping that results in a successful capturing of the prey by the team of predator agents. However, as the preliminary results indicated, the predators that use a straightforward sensor could not resolve more than just few of the tested initial situations. Thus, to improve the generality of the evolved behaviour, we proposed an asymmetric sensory morphology of predators—an angular offset to the sensor relative to the longitudinal axis—and coevolved the amount of such an offset together with the behaviour of predators. The behaviours, coevolved with a sensor offset between 12° and 38°, resulted in both an efficient and consistent capture of the prey in all tested initial situations. Moreover, some of the behaviours, coevolved with sensor offset between 18° and 24°, demonstrated a good generality to the increased speed of the prey and a good robustness to perception noise. The obtained results could be seen as a step towards the engineering of asymmetric small-scale for delivery of medicine, locating and destroying cancer cells, microscopic imaging, etc.

## 1. Introduction

With the advancement of technology and invention of the optical and electric microscopes, the humanity started exploring the miniature world. With these new discoveries; however, new problems started to arise. To discover the solutions to them, humankind turned to creating micro- and nanomachines on their own [1]. As a species, striving to survive various lethal conditions, we are exposed to the most prominent field of use for these new nanomachines, medicine. There are many procedures that are hard to perform by a human medical doctor and for which the newly created microrobots are perfectly suited [2]. Such procedures, in which the traditional approaches could harm the surrounding (healthy) body tissues, include brain surgery, video diagnostics in hard-to-reach places, and pinpoint drug delivery (much needed in chemotherapy). Some of the advantages that nanotechnology provides are continuous monitoring, rapid response to a sudden change in conditions, minimal trauma to the tissues, relatively short recovery time, and minimal posttreatment care [3].

In our research, we are employing a multiagent system (MAS) as the model of a team of such simple small-scale robots. The advantage of the developed MAS, compared to centralized systems with analogical functionalities, is that it offers an increased modularity, reduced complexity (offering an intuitive solution to the divide-and-conquer approach of developing and deploying complex software systems), and flexibility to diverse software and hardware platforms. From the viewpoint of the end-users, the benefits of using MAS are the superior robustness, increased fault tolerance, scalability, and performance. The latter is especially true, as the MAS could solve (inherently parallel or distributed) problems much faster than centralized (or single agent) systems. Moreover, due to their complex, nonlinear nature, MAS could often solve problems that a single agent is unable to solve. The whole team of multiple agents is expected to exhibit a behaviour that can be regarded as an emergent (high-level) property of the much simpler (lower-level) properties of the agents, or as a whole that is “more than the sum of its entities” (Aristoteles, 384 a.C.-322 a.C.), and, therefore, could not be devised by applying the conventional top-down software engineering approaches.

Currently, there are various challenges that are slowing the progress of the real-world applicability of MAS modelling the societies of small-scale robots. One of these challenges stems from the very advantage of these robots—their small size. The physical constrains imply that these robots could not feature a complex morphology—both the sensors and moving mechanisms need to be very simple to be able to fit in the limited space of the bot's body. The robots would be behaviourally simple too, in that their decision-making would involve no computing, but rather a direct mapping of the (few) perceived environmental states into corresponding commands to their actuators. Most likely, the communication (if any) between the individual agents would be impossible to be realized in a direct manner and would be fulfilled implicitly, through the corresponding changes in the environment. Such robots can be regarded as an ultimate case of Occam's razor principle, applied both to their morphology and decision-making. Such simplicity further widens the gap between the available properties of the individual robots and the desired complex overall behaviour of the team of such robots as a whole. This is especially true in our case as we focus on creating bots that could traverse the human body autonomously, rather than being guided by an external force and continuously monitored [4, 5].

The factors in favour of the possible small-scale implementation of the robots considered in our work include (i) their *minimalistic implementation*, (ii) compatibility with the *fluid dynamics* at very low Reynolds numbers, and (iii) robustness of the behaviour of the robots to *Brownian collisions* (diffusion), as elaborated below.

The *minimalistic implementation* implies very simple sensors, control, and effectors of the robots. Indeed, the single line-of-sight *sensor* adopted in our work is seen as one of the examples of “extreme simplicity” in robot (agent) perceptions in the fields of multiagent systems and swarm robotics [6–11]. It could be implemented by a single (or just a few) receptor(s)—pixel (of a camera), nanoparticle, etc. The control is also very simple—a purely reactive, direct mapping of the (few) perceived environmental states into corresponding effectors' commands. The effectors are modelled as wheels in our robots, arranged in a differential drive configuration, which is seen as the minimal configuration for robots in 2D environments that allow both linear movement and rotational (steering) movement of the robots [6–11].

Two of the features of the wheels of the robots considered in our work are related to their compatibility with *the fluid dynamics* at very low Reynolds numbers, pertinent to the real world of small-scale robots: (i) the wheels control the movement of robots by the resulting vectors of linear velocities (rather than forces that would require the consideration of torque of motors, mass and rotational momentum of the robots, resulting acceleration, etc.) applied to each of the two sides of the robot and (ii) the changes in these velocities occur instantly (there is no coasting). We introduced these features—controlling the robot by linear velocities of wheels that change instantly—to bridge the reality gap between the model of our robots and the very low Reynolds number dynamics of the small-scale robots. Indeed, at very low Reynolds numbers, the movement of the robot is characterized by the dominance of the viscous forces over the inertial ones [12]. Consequently, the mass (and the inertia) is not a relevant factor in such a movement, and the changes of velocities of the small-scale robots would happen almost instantly. In an eventual 3D implementation, the wheels could be superseded by more general “thrusters” that model the actual propulsion source of the small-scale robots—e.g., bioinspired rotating helical flagellum and a rotary artificial molecular machine [11, 12].

The robustness to *Brownian collisions* could be achieved by just having a sufficiently large size of the robot [12]. From another perspective, as we shall elaborate later, we tested the robustness of the proposed MAS to perception noise. The effects of collision with particles subjected to Brownian motion is somehow different from just a perception noise; however, our experiment could be seen as a first step towards the verification of the system in highly dynamic, uncertain environments.

Gauci et al. [6] previously modelled similar simple robots as agents. The agents were able to self-organize in order to solve the simple robot aggregation problem. The same framework was also successfully applied for the more complex object-clustering problem [7] in which the agents need to interact with an additionally introduced immobile object. The very possibility of a team of such agents to conduct an elaborate social (surrounding) behaviour in an environment featuring dynamic objects was recently demonstrated by Ozdemir et al. [8] in solving the shepherding problem, where a team of simple agents (shepherds) needs to guide multiple dynamic agents (sheep) toward an a priori-defined goal.

In our study, we proposed the use of a similar team of simple agents for the solution of a different task—the well-studied, yet difficult to solve predator-prey pursuit problem (PPPP) [13–17]. In this PPPP, eight identical, simple agents (predators) are used to capture the single dynamic agent (prey).

Our objective is to investigate the feasibility of applying the genetic algorithms (GA) to evolve such direct mapping of the four perceived environmental states into respective velocities of the wheels of predator agents that result in a successful capture of the prey by the team of predator agents. Moreover, we are interested in whether coevolving (i) the asymmetric sensory morphology—an angular offset of the sensors—of predator agents and (ii) their behaviour would result in more efficient and general capturing.

Our motive for using the proposed instance of PPPP is based on the increased complexity of the problem, compared to the previously studied tasks [6–9]. In comparison to the previously investigated domains, PPPP requires the agents to exhibit a more diverse behavioural set, including exploration of the environment and surrounding and capturing the prey. In contrast to [8], in our implementation of the PPPP framework, the emergence of such behaviours is made additionally complicated, by the introduced constrains to the sensory and moving abilities of the predator agents. Compared to the unlimited range, assumed in other works, our predators feature myopic, limited-range sensors, and their movement speed is equal to that of the prey, instead of being faster [6, 8]. Furthermore, the initial position of the predators is such that the prey is not being surrounded, which may ease the task of capturing it. This can be viewed as injecting the clustered team of small-scale robots at a certain point into the human body.

An additional motivation of our research is the recognition that while many real-world scenarios could be, indeed, reduced to the previously researched wall-following, dispersal [9], clustering [6], and shepherding problems [8], there would be few scenarios—requiring a direct physical contact with an active prey—that could be modelled by the proposed instance of PPPP [18–20]. These scenarios might include pinpoint drug delivery, surrounding and destroying (cancer) cells or bacteria, gathering around cells to facilitate their repair or imaging, etc.

The remainder of this article is organized as follows. The second section describes the entities in the PPPP. In the third section, we elaborate the GA, adopted for the evolution of predator behaviours. In the fourth section, we present the experimental results and introduce the proposed asymmetric sensory morphology of predators. In the same section, we show the results on the robustness and generality of the evolved predator agents. The fifth section discusses the advantages of asymmetric morphology and the emergent behavioural strategies of the predator agents. We draw a conclusion in the sixth section.

## 2. Entities

### 2.1. Predator Agents

Each of the eight (identical) predator agents models a simple cylindrical robot with a single line-of-sight sensor featuring a limited range of visibility and two wheels (controlled by two motors) in a differential drive configuration.

**Figure 1:**
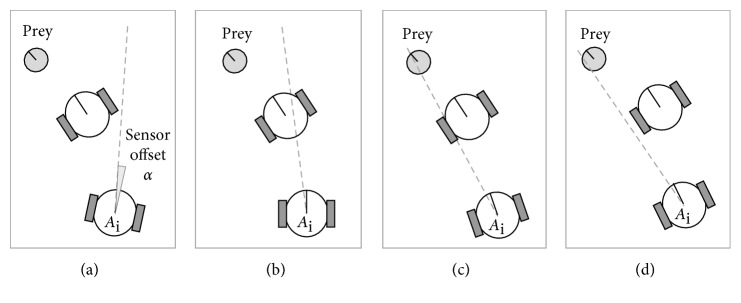
The four possible environmental states that are perceived by any given predator agent. (a) State <00>. (b) State <10>. (c) State <11>. (d) State <01>.

The single line-of-sight (beam) sensor provides two bits of information, where each bit encodes whether an entity—either a (nearest) predator agent or the prey, respectively—is detected (if any) in the line-of-sight within the limited range of visibility. The implementation of such sensor would consist of two photodetectors, sensitive to two different, nonoverlapping wavelengths of (ultraviolet, visible, or infrared) light reflected (or emitted) by predators and prey, respectively. Each of these two photodetectors provides one bit of information. Equipped with such sensors, the predators could perceive only four discrete possible states—<00>, <01>, <10>, and <11>, as shown in Figure 1—of the environment. The state <11> is the most challenging one to perceive. It could be sensed, however, under the following assumptions: (i) the prey is taller than the predators and (ii) to not obscure the shorter predators, the cross-section of the prey is either much narrower than predators or (at least partially) transparent for the light to be perceived by the predators. Notice that the perceived environmental states do not provide the predators with any insight about the distance to the perceived entities, nor their total number.

In our previous work [21], we noticed that the classical morphology of the agents—in which sensor is aligned with the longitude axis of the agents—results in successful solutions of more than a few initial situations. Therefore, instead of the commonly considered straightforward orientation of the sensor of the predators, we proposed an angular (e.g., counterclockwise) offset relative to their longitudinal axis. We speculated that such an asymmetric sensory morphology would allow the predators to evolve a more efficient capturing behaviour by implementing an equiangular (proportional) pursuit of the prey, aiming at the (estimated) point of the contact with the moving prey, rather than the currently perceived position of the latter. The proposed asymmetric morphology does not compromise the intended simplicity of the agents. The main features of the agents, used during the evolution of the behaviour of prey agents, are summarized in Table 1.

The entirely reactive behaviour of the predator agents could be described as a direct mapping of each of the perceived environmental states into a corresponding rotational speed of the wheel motors. For simplicity, instead of mapping into rotational speeds (e.g., RPM) of the motors, we will assume a mapping into the linear velocities of the wheels, expressed as the percentage—within the range (−100%,…, +100%)—of their respective maximum linear velocities (10 units/s, as shown in Table 1). For example, a velocity of −20% implies that the motor of the wheel is rotating at 20% of its maximum linear velocity, and the wheel propels the corresponding side of the robot in a backward (negative) direction with a linear speed of 2 units/s (i.e., 20% of the maximum linear speed of the wheel). The purely reactive decision-making of the predator agents could be formally defined by the following octet:

(1) $$\begin{array}{c} A=\left\{V_{00\text{L}},V_{00\text{R}},V_{01\text{L}},V_{01\text{R}},V_{10\text{L}},V_{10\text{R}},V_{11\text{L}},V_{11\text{R}}\right\}, \end{array}$$

where *V*_00L_ and *V*_00R_ are the linear velocities (as a percentage, set within the range (−100%,..., +100%), of the maximum linear velocity) of the left and right wheels of the agents for the perceived environmental state <00>, while *V*_01L_, *V*_01R_, *V*_10L_, *V*_10R_, *V*_11L_, and *V*_11R_ are analogical velocities for the perceived environmental states <01>, <10>, and <11>, respectively.

Our objective of coevolving (via GA) the behaviour and asymmetric sensory morphology of the agents could be rephrased as coevolving (i) such values of the velocities, shown in the octet in equation (1), together with (ii) the angular offset of the sensor, resulting in an efficient capturing behaviour of the team of predator agents. We shall elaborate on such a coevolution in the next section.

### 2.2. Prey

The prey is equipped with an omnidirectional sensor, with limited visibility range. To balance the advantage that the omnidirectional sensor gives to the prey, compared to the single line-of-sight sensor of the predators, the viewing distance of the prey is only 50 units, compared to the 200 units of the predators. The maximum speed of the prey, however, is identical to that of the predators. These conditions would encourage the predator agents to evolve cooperative behaviours as they will be unable to capture the prey alone. Another viewpoint suggests that a successful solution to PPPP, defined in such a way, could demonstrate the virtue of the MAS as it could solve a problem that a single (predator) agent could not.

In contrast to the predator behaviours, we implemented a handcrafted behaviour for the prey. The prey attempts to escape from the closest predator (if any) by running at its maximum speed in the direction that is exactly opposite to the bearing of the predator. The prey remains still if it does not detect any predator. Table 1 shows the main features of the prey agent.

### 2.3. The World

**Figure 2:**
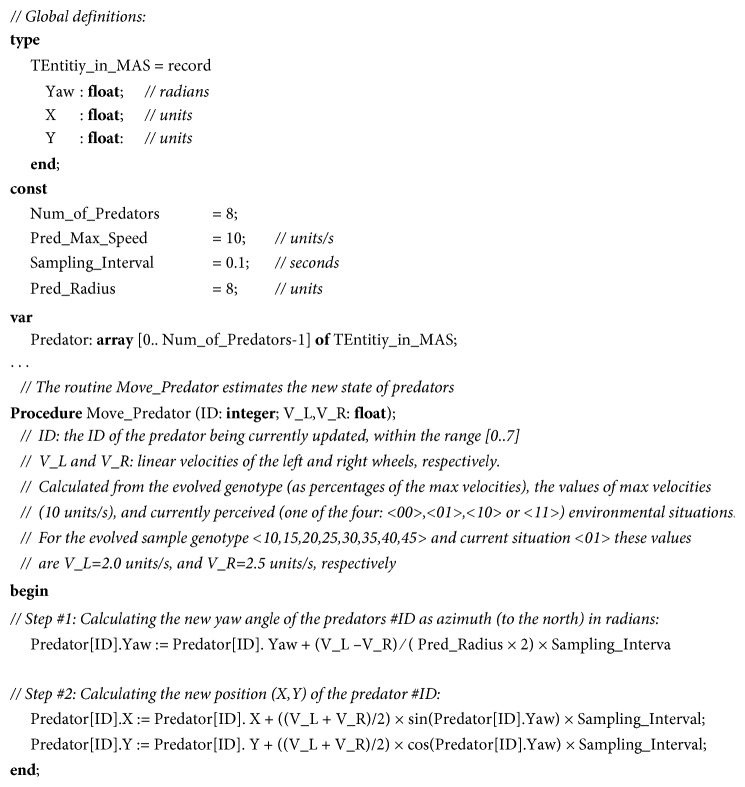
The pseudocode of estimating the new state of the moving predators.

We modelled the world as a two-dimensional infinite plane with a visualized part of 1600 × 1600 units. We update the perceptions, decision-making, and the resulting new state (e.g., location, orientation, and speed) of agents with a sampling interval of 0.1 s. The duration of trials is 120 s, modelled in 1200 time-steps. We approximate the new state of predators in the following two steps, as illustrated in Figure 2. First, from the current orientation, the yaw rate, and the duration of the sampling interval, we calculate the new yaw (orientation) angle (as an azimuth t the north) of the agents. The yaw rate is obtained from the difference between the linear velocities of the left and right wheels, and the length of the axis between the wheels. Then, we calculate the new position (i.e., the two-dimensional Cartesian coordinates) as a projection (in time, equal to the duration of the sampling interval) of the vector of the linear velocity of predators. The vector is aligned with the newly calculated orientation, and its magnitude is equal to the mean of the linear velocities of the two wheels.

## 3. Evolutionary Setup

We decide to apply a heuristic, evolutionary approach to the “tuning” of the velocities of both wheels for each of the perceived four environmental situations because we are a priori unaware of the values of these velocities that would yield a successful behaviour of the team of predator agents. As we briefly mentioned in Section 1, MAS, as a complex system, features a significant semantic gap between the simple, hierarchically lower-level properties of the agents and the more elaborate, higher-level behaviour of the whole system. Consequently, we would be unable to formally infer the values of the octet of velocities of the wheels of agents from the desired behaviour of the team of such agents. Similarly, we are unaware of the value of the angular offset of the sensor, resulting in an efficient capturing behaviour of the agents. Moreover, the values of velocities of both wheels and the value of the angular offset of the sensor would, most likely, be dependent on each other.

Alternatively, in principle, we could have adopted another, deterministic, approach, such as, for example, a complete enumeration of the possible combinations of the eight velocities of wheels and the sensor offset. If each of these 8 velocities is discretized into, say, 40 possible integer values ranging from −100% to +100% and the sensor offset just into 20 values, then the size of the resulting search space would be equal to 40^8^ or about 1.3 × 10^14^. This would render the eventual “brute force” approach, based on complete enumeration of possible combinations of values of velocities computationally intractable.

As an alternative to the brute force search, we could apply reinforced learning (RL) in order to define the good mapping of the four perceived environmental states into the four pairs of velocities of wheels. However, MAS are complex, nonlinear systems, and there is a significant gap between the properties of the entities and the (emergent) properties of the system. RL would obtain a “reward” from the system (i.e., the efficiency of the team of predators) and will try to modify the properties (the four pairs of velocities of wheels) of the entities. Due to the complexity and nonlinearity of MAS, this is not a straightforward task. This is also related to the intra-agent credit-(or blame-) assignment problem, as we could not tell which part of the agents is responsible (and therefore–should be modified) for the bad overall behaviour of the system.

Evolutionary computing solves these challenges in an elegant way, by obtaining the fitness value from the system, as a whole (i.e., the efficiency of predators in capturing the prey) and then modifying the properties of entities (pairs of velocities of wheels of predators) via genetic operations, crossover and mutations.

Yet another challenge in RL is the delayed reward problem—the success (if any) of the system (team of predators) would occur several hundred time-steps into the trial, but might be related to the earlier behaviour phases of the team of predators—such as the dispersing (exploration of the environment) at the very beginning of the trial. Regarding the delayed reward problem, the evolution, as a holistic approach, does not care about how to achieve the success, but rather about the overall (final) outcome of the trial.

**Figure 3:**
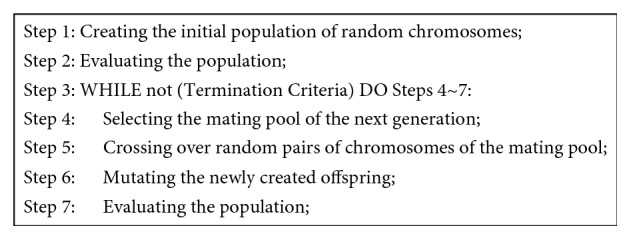
The main algorithmic steps of the GA.

In our work, we apply GA, a nature-inspired heuristic approach that gradually evolves the values of a set of parameters in a way similar to the evolution of species in nature. GA has proved to be efficient in finding optimal solution(s) to combinatorial optimization problems featuring large search spaces [22–24]. Thus, consonant with the concept of evolutionary robotics [25], we adopted GA to evolve the values of the eight velocities of the wheels and the offset of the sensor that result in an efficient behaviour, presumably involving exploring the environment and surrounding and capturing the prey, of the team of predators. The main algorithmic steps of the adopted GA are shown in Figure 3, and its main attributes, genetic representation, genetic operations, and fitness function, are elaborated below.

### 3.1. Genetic Representation

We genetically represent both (i) the decision-making (behaviour) of the predator agents and (ii) their sensory morphology in a single “chromosome”. The latter consists of an array of eight integer values of the evolved velocities of wheels of the agents and an additional allele encoding the angular offset of their sensor. The values for the velocities are constrained within the range (−100%…+100%) and are divided into 40 possible discreet values, with an interval of 5% between them. The angular offset is defined in range between 2° and 40°, counterclockwise, divided into 20 possible discreet values, with an interval of 2° between them. The decided number of discrete values (and the interval between these values, respectively) provides a good trade-off between the precision of “tuning” (i.e., expressiveness of the genetic representation) and the size of the search space of GA. The population size is 400 chromosomes. The breeding strategy is homogeneous in that the performance of a single chromosome, cloned to all predators is evaluated.

### 3.2. Genetic Operations

Binary tournament is used as a selection strategy in the evolutionary framework. It is computationally efficient and has proven to provide a good trade-off between the diversity of population and the rate of convergence of the fitness. In addition to the tournament selection, we also adopted elitism in that the four best-performing chromosomes survive unconditionally and are inserted into the mating pool of the next generation. In addition, we implemented, with equal probability, both one- and two-point crossover. The two-point crossover results in an exchange of the values of both velocities (of the left and right wheels, respectively) associated with a given environmental state. This reflects our assumption that the velocities of both wheels determine the moving behaviour of the agents (for a given environmental state), and therefore, they should be treated as a whole—as an evolutionary building block. Two-point crossovers would have no destructive effect on such building blocks. The one-point crossover is applied to develop such building blocks (exploration of the search space), while the two-point crossover is intended to preserve them (exploitation).

### 3.3. Fitness Evaluation

**Figure 4:**
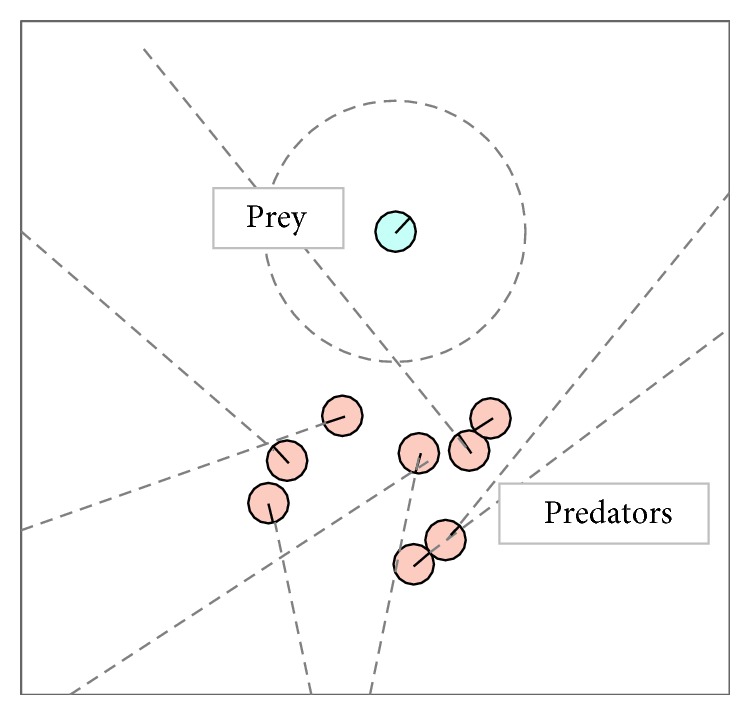
A snapshot of a sample initial situation.

Our aim is to coevolve the behaviours and sensory morphology of the team of predators that are general to multiple initial situations, rather than a behaviour that is specialized to a particular one situation. To facilitate such an evolution, we evaluated each of the evolving chromosomes in 10 different initial situations. In each of these situations, the prey is located in the centre of the world. The predators are scattered in a small cloud situated south of the prey. A snapshot of a sample initial situation is shown in Figure 4. The distance of the cluster, of agents, to the prey is calculated as follows: ID of the current situation × 2 + (random of 50 units). This helps reduce the impact of the first few evolutionary runs, when the predators are learning how to move around the environment to find the prey.

The overall fitness is the sum of the fitness values, scored in each of the 10 initial situations. For a successful situation (i.e., the predators manage to capture the prey during the 120 s trial), the fitness is equal to the time needed to capture the prey. If the initial situation is unsuccessful, the fitness is calculated as a sum of (i) the closest distance, registered during the entire trial, between the prey and any predator and (ii) a penalty of 10,000. The former component is intended to provide the evolution with a cue about the comparative quality of the different unsuccessful behaviours. We verified empirically that this heuristic quantifies the “near-misses” well and correlates with the chances of the predators—pending small evolutionary tweaks in their genome—to successfully capture the prey in the future. The second component is introduced with the intension to heavily penalize the lack of success of predators in any given initial situation.

Our PPPP is an instance of a minimization problem, as lower fitness values correspond to better performing team of predator agents. Since we are aiming to discover the best possible solution to the problem, no target fitness value is incorporated in the termination criterion of the evolution. Instead, this criterion includes the following two conditions: the number of the evolved generations is equal to 200 or the best fitness remains unchanged (stagnated) for 32 consecutive generations.  Table 2 shows the main parameters of the adopted GA.

## 4. Experimental Results

### 4.1. Evolving the Team of Straightforward Predator Agents

**Figure 5:**
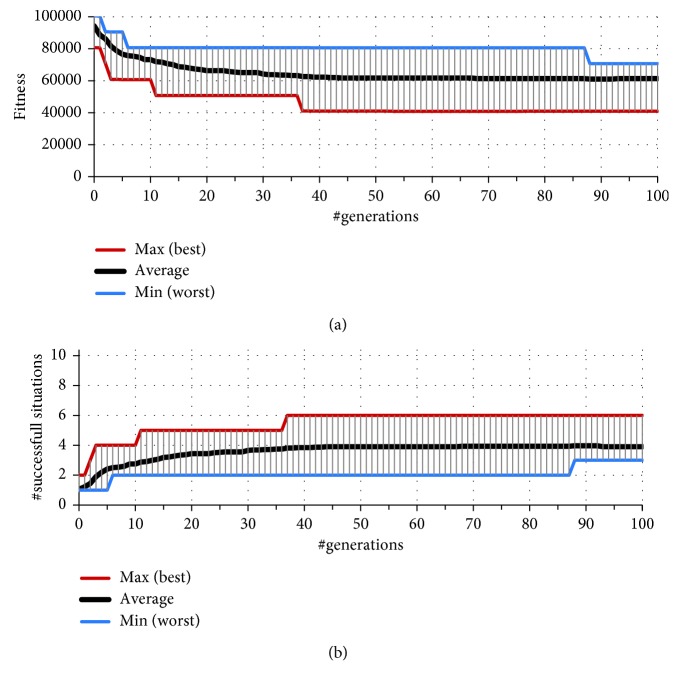
Convergence of the values of best fitness (a) and the number of successful situations (b) of 32 independent runs of GA evolving the behaviour of straightforward (without an offset of the sensor) predator agents.

The experimental results of 32 independent runs of the GA evolving only the behaviour of the predator agents are illustrated in Figure 5. In these runs of the GA, the sensory morphology of predators was fixed, and the sensor offset was set to 0. As Figure 5(a) illustrates, the mean value of the fitness slowly converges to approximately 60,000, indicating that, on average, only 4 (of 10) initial situations could be successfully resolved (Figure 5(b)). The best result, achieved by the evolved team of predators, is only 6 successful situations. These results suggest that the instance of PPPP featuring predators with straightforward sensors is, in general, intractable.

### 4.2. Coevolving the Asymmetric Morphology and the Behaviour of Predator Agents

**Figure 6:**
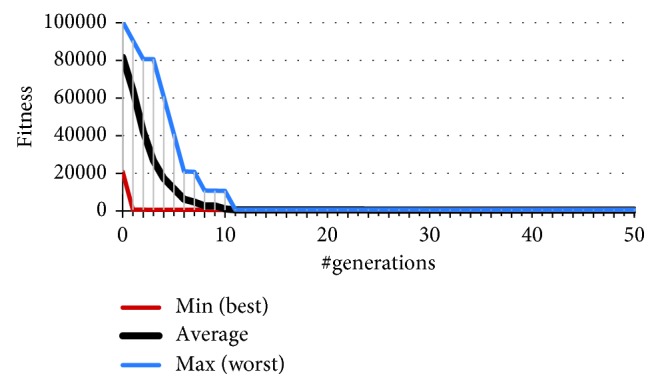
Convergence of the best fitness of 32 independent runs of GA.

**Figure 7:**
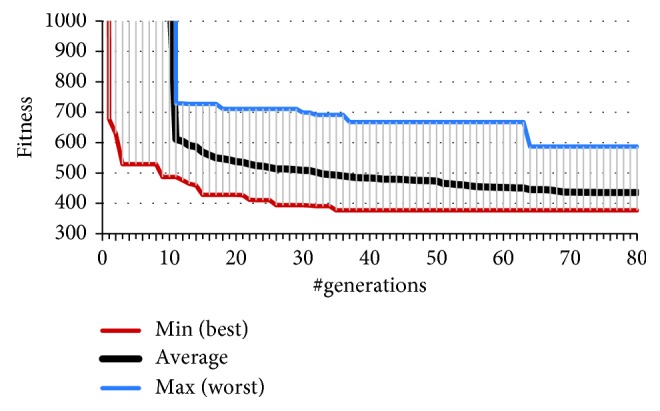
A more detailed illustration of the convergence of the best fitness of 32 independent runs of GA.

**Figure 8:**
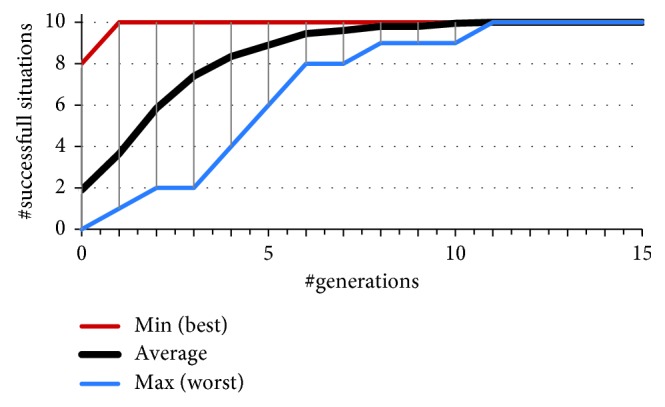
Convergence of the number of successful situations of 32 independent runs of GA.

As Figures 6–8 illustrate, just by adding the offset, the results in number of successful initial situations and overall fitness significantly improve compared to the evolution of the team straightforward predator agents featuring no angular offset of sensors. On average, the predators were able to resolve all 10 initial situations by 10th generation of the GA. From all 32 independent runs of GA, there is one distinguished solution (from now on we will refer to it as the fastest evolved solution SFE) which successfully solves 8 (of 10) in the first generation. The chromosome of this solution encodes for offset of the sensor of 20°. This confirms the findings in our previous research [21, 26] that a team of predators with 20° sensor offset yields favourable results during evolution. As we will discuss later, this is also true in case of additional, unforeseen, situations and presence of perception noise. However, from all 32 solutions, this is not the one that has achieved the best overall fitness value. The best behaviour of agents (manifested by the achieved lowest of fitness value) was obtained by the solution SBF featuring a sensory offset of 16°. Compared to the fastest evolving solution SFE, the solution SBF evolved a bit slower and solved all 10 situations by 6th generation, achieving the terminal fitness of 369 (compared to 417 of solution SFE).

**Figure 9:**
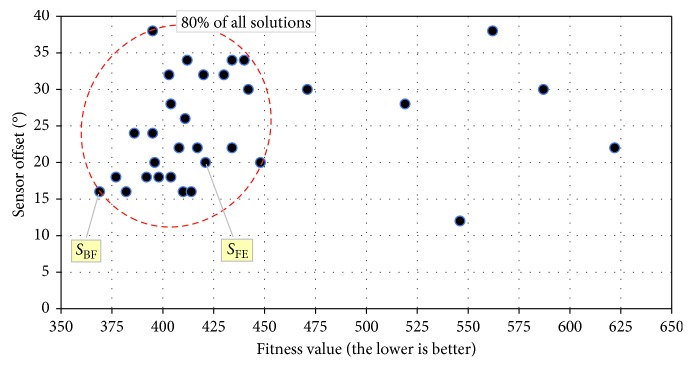
Sensor offset and the fitness value of all 32 solutions obtained form 32 independent runs of the GA. The fastest evolved and the best overall solutions are denoted as solutions SFE and SBF, respectively.

Figure 9 illustrates the angular offset of the best solutions obtained from each of the 32 independent runs of the GA. As seen in Figure 9, the fitness of 80% (i.e., 26 of 32) of solutions is in the range between 369 and 448, i.e., the team of agents could capture the prey (on average over all 10 situations) between 36.9 s and 44.8 s into the allocated 120 s of the trial. The fitness of the worst solution is 622, meaning that the team of predator agents captures the prey, on average, at 62.2 s, i.e., around the middle of the 120 s trial. Moreover, as Figure 9 illustrates, for a particular value of the sensor offset, there are multiple solutions with different fitness values, meaning that there are variations in the behaviour of the morphologically identical predators and that the sensory asymmetry is only a precondition for an efficient capturing behaviour of the predators. Analogically, very similar fitness values could be achieved by predators featuring different sensor offset, suggesting that the combination of both (i) the morphology and (ii) the behaviour, rather than a particular instance of each of them, is important for the success of the behaviour of predator agents.

**Figure 10:**
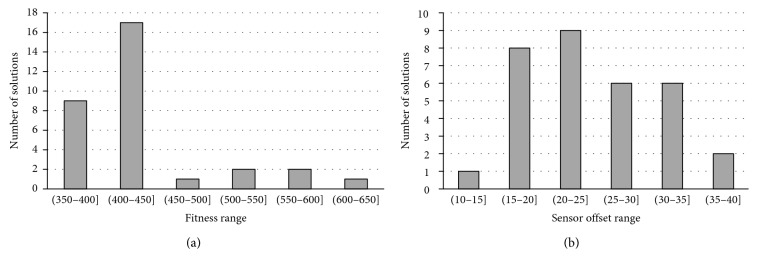
The breakdown of the number of the successful situations (a) and the sensor offset (b) of all 32 solutions obtained form 32 independent runs of the GA.

The breakdown of the number of the successful situations and the sensor offset of all 32 solutions are illustrated in Figure 10. As depicted in Figure 10(a), the sensor offset of 90% (i.e., 29 of 32) of solutions is within the range (15°,…, 35°). There is no evolved solution that features a sensor offset lower than 10°, which confirms experimentally our initial hypothesis about the beneficial effect of the asymmetric morphology of predators on the efficiency of their behaviour. The statistical characteristics of all 32 solutions are shown in Table 3.

### 4.3. Generality of the Evolved Solutions

**Figure 11:**
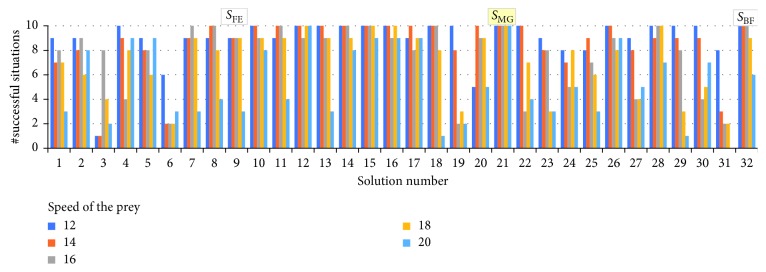
The number of successfully solved situations by the evolved 32 solutions for the speed of prey being increased from 10 to 12, 14, 16, 18, and 20 units/s, respectively.

**Figure 12:**
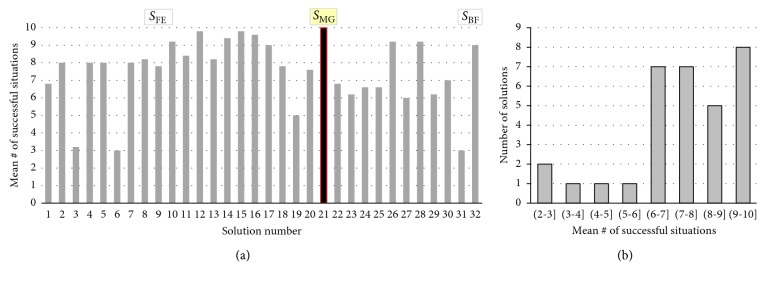
Generality of the evolved 32 solutions to the changes in the speed of prey from 10 to 12, 14, 16, 18, and 20 units/s: the mean number of successfully solved situations (a) and its breakdown (b).

To assess the generality of the evolved behaviour of the predator agents, we will examine how their performance (i.e., the number of successfully resolved initial situations) degrades with the increase of the speed of the escaping prey. We tested all 32 solutions, obtained via the GA (for the speed of the prey equal to 10 units/s), for speeds of the prey, unforeseen during the evolution, of 12, 14, 16, 18, and 20 units/s, respectively. The number of initial situations successfully solved by each of the 32 solutions for each of the considered speed of the prey is shown in Figure 11. The mean (over the whole range of speeds of the prey) of the successfully solved situations by each of these solutions, and its breakdown are depicted in Figure 12. As these figures illustrate, one of these solutions, denoted as *S*_MG,_ is most general in that it features no degradation in the number of successful situations with the increase of the speed of the prey. Moreover, its fitness value remains under 500 (i.e., the agents capture the prey earlier than 50 s into the 120 s trial) for all considered speeds of the prey. As shown in Table 4, the sensor offset of *S*_MG_ is 24°.

### 4.4. Robustness to Perception Noise

**Figure 13:**
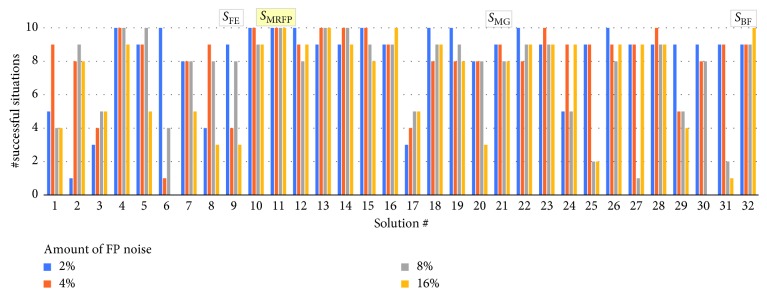
Robustness to FP noise of each of the 32 evolved solutions.

**Figure 14:**
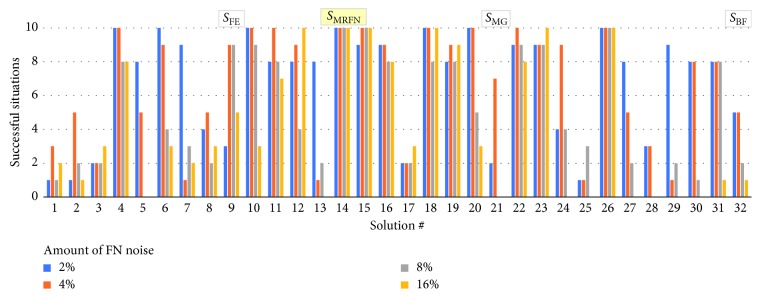
Robustness to FN noise of each of the 32 evolved solutions.

We evaluated the robustness of the 32 evolved solutions, evolved in a noiseless environment, to a random perception noise. We introduced two types of noise—a false positive (FP) and a false negative, respectively. The former results in either of the two bits of perception information to be occasionally (with a given probability) read as “1” regardless of whether an entity is detected in the line-of-sight of the predators or not. False negative noise (FN) results in readings of “0” even if an entity is seen. We focused on these types of noise as we assume that the perception subsystem of predators, yet being rather simple, would require an appropriate thresholding of the sensory signal. A combination of unfavourable factors, such as incorrectly established threshold and variable noise levels in the environment or in sensors, would result in the considered two types of perception noise. Figures 13 and 14 show the degradation of the number of successfully solved situations by all 32 solutions for different amount of FP and FN perception noise, respectively.

As Figures 13 and 14 illustrate, neither the fastest evolved solution *S*_FE_ nor the solution with the best fitness *S*_BF_, which we previously discussed, features a good robustness to perception noise. On average, they solve 6.25 initial situations each, with the introduction of either FP or FN noise. Both solutions yield similar results with the difference between them being that *S*_BF_ is more robust to FP noise while *S*_FE_ is better in case of FN noise. Instead, the solutions *S*_MRFP_ and *S*_MRFN_ (featuring a genotype as shown in Table 4) emerge as most robust to FP noise and FN noise, respectively. Solution *S*_MRFP_ manages to solve the tests with FP noise perfectly, while maintaining satisfactory performance in the tests with FN noise, being able to solve on average 8.25 initial situations, depending on the level of FN noise. On the contrary, the agents controlled by *S*_MRFN_ solve the situations with FP noise perfectly, while being able to solve an average of 9.5 initial situations in the situations with FN noise, resulting in the best overall performance. The sensor offset of *S*_MRFP_ and *S*_MRFN_ is 18° and 20°, respectively (Table 4).

## 5. Discussion

### 5.1. Advantage of Asymmetric Morphology

We have shown that introducing an angular offset to the viewing sensor facilitates a more effective behaviour of the team of agents and increases the efficiency of evolution of such behaviour. The experimental results suggest that the behaviour, evolved with a sensor offset of 20° (in solution *S*_MRFN_), is most robust to noise and is close enough in terms of fitness to the best-performing team of agents in noiseless environments. The fitness of *S*_MRFN_ is 421 compared to 369 of *S*_BF_. While *S*_MG_ shows best results in the generality test, with perfect score in all initial situations, it falls short in the noise robustness test. This leads us to believe that *S*_MRFN_ is an example of a good combination of coevolved behaviour and asymmetric morphology of the predator agents. On average, *S*_MRFN_ manages to solve 9.57 and 9.65 situation in the generality and robustness tests cases. The angular offset of 20° of *S*_MRFN_ provides a good trade-off between the tangential and radial (i.e., towards the prey) components of the speed vector of the chasing predators.

**Figure 15:**
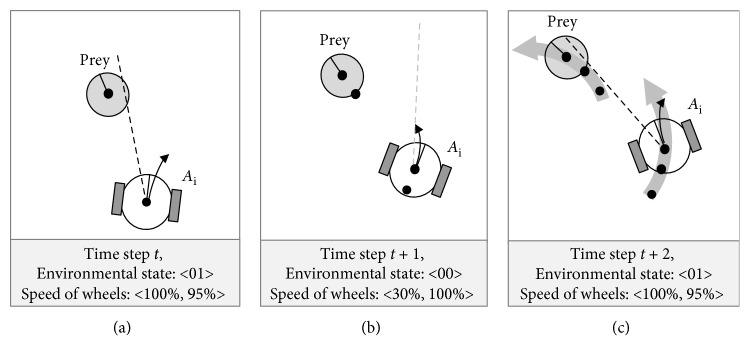
Chasing the prey by a sample predator agent A_*i*_.

The beneficial effect of the sensor offset is that it helps the chasing predator to implicitly determine the position of the prey if the latter disappears. Having a counterclockwise displacement means that most of the time the disappeared prey, due to the parallax induced by the movement of the predator, would be to the left, and consequently, a slight turn to the left would allow relocating it again. Therefore, one of the virtues of the sensor offset is in the *more deterministic direction* of the disappearance of the prey, almost certainly to the left, which in turn facilitates a faster rediscovery and consequently, a more reliable tracking of the latter by the predator. Moreover, as shown in Figure 15, the chase by the predator featuring an asymmetric morphology would result in a characteristic *circular trajectory* of both the predator and the prey. With the rather challenging but realistic assumption that initially the prey is not being surrounded by the predators (as illustrated in Figure 4), such circular trajectories would facilitate the surrounding as the prey would be shepherded (driven) by a single predator towards the pack of the remaining predators.

### 5.2. Emergent Behavioural Strategies

**Figure 16:**
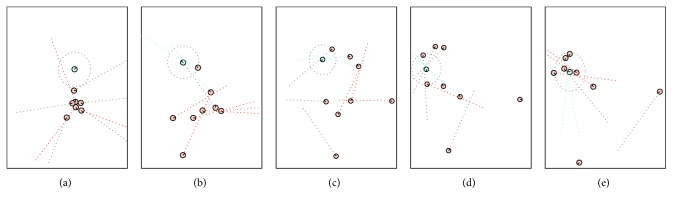
Emergent behavioural phases exhibited by the team of predator agents: exploring (a), shepherding (b–d), and capturing (e).

Following our previous work on coevolving behaviour and morphology [27], in this section, we review the behavioural strategies, emerging from the team of agents controlled by the evolved solution that is most robust to noise, the solution has the greatest success rate, the solution *S*_MRFN_. The values of the evolved velocities of motors and the sensor offset are shown in Table 4. The team of predator agents manifests the following three types of behaviours, executed in three consecutive phases of the trial: (i) e*xploring* the environment by distancing themselves from each other (controlled by velocities *V*_10_) or circling around until they find a peer or the prey (*V*_00_), (ii) *shepherding* (driving) the prey (by some of the predators) in an circular trajectory (*V*_01_), and (iii) *capturing* the prey (*V*_11_).  Figure 16 illustrates the different phases the agents go through in the process of catching the prey. A video of how the team of predators deals with all 10 situations can be found at http://isd-si.doshisha.ac.jp/m.georgiev/2018-12-08-SA20deg.mp4.

As shown in Figure 16(a), in the beginning, all agents have no vision of either the prey or any of the peers. Following the mapping of *V*_00L_ = 30% and *V*_00R_ = 100%, they start turning around in a circular motion—scanning the environment in an attempt to find another entity. Detecting a peer activates the set of velocities *V*_10L_ = −75% and *V*_10R_ = −70%, which forces the predators to rapidly move away from the perceived agent, which facilitates a better dispersion and a coverage of a wider area. This enhances the ability of the predators to *explore* the environment and to discover the prey. The second stage begins when any of the predators discovers the prey. The mapping *V*_01L_ = 100% and *V*_01R_ = 95% results in moving forward at highest speed and slightly turning to the right, which helps keeping the prey always in the same relative position to the agent, to the left side, as shown in Figures 15 and 16(b)–16(d). Once the prey becomes invisible, as shown in Figure 16(b), the predator exhibits an *embodied cognition* that the disappearance is a result, in part, of its own forward motion; therefore, the new location of the prey is, due to the counterclockwise offset of the sensor, most likely on the left of its own orientation. The evolved *V*_00L_ = 30% and *V*_00R_ = 100% are activated (Figure 15(c)) resulting in a circular motion to the left, until the agent rediscovers the disappeared prey. Moreover, as Figures 16(b)–16(d) show, a single predator, due to its sensor offset, *shepherds* (*drives*) the prey in a circular, counterclockwise trajectory into the (already dispersed) other predators. The final behavioural phase begins with the surrounding of the prey from all sides of the world by both thus far and newly encountered chasing predators, as illustrated in Figure 16(e). When approaching from opposite sides, the predators are able to see both the prey and a peer, which activates the mapping *V*_11L_ = 100% and *V*_11R_ = 100%. Since they have a slight angular offset, it is possible for only two predators to catch the prey, as illustrated in Figure 16(e). One of the predators chases the prey from behind and guides it to its frond left side, while the other intercepts it from the exactly opposite direction.

At the same time, we can see in Figures 16(d) and 16(e) that two of the agents keep distancing themselves from the group. The agents seem to exhibit an emergent knowledge [28] that not all eight agents are needed to capture the prey. For the group of agents to be successful, the most important mission is to capture the prey, rather than which particular agent does it. As the performance of the predators is calculated based on the success of the group instead of that of the particular individual agent, such behaviour helps the team (as a whole) by expanding the search field and finding the prey faster, especially when it is further away from the predators. If, instead, the agents were trying to find the prey and capture it by themselves via “greedy chase,” they would inevitably fail because (i) the prey is fast enough to run away from a single predator and (ii) the predators would have been unable to engage in any organized behaviour that allows surrounding and ultimately, capturing the prey.

The most significant difference between the evolved behaviour of straightforward predator agents and that of the agents with asymmetric morphology is in the second behavioural phase, *shepherding*. This phase could not be observed in the behaviour of the straightforward agents. At the same time, as we elaborated above, it plays an important role in the successful capturing of the prey.

### 5.3. Heterogeneous vs. Homogeneous Systems

During our research, we considered a different configuration for the multiagent system featuring several types of predator agents where each of them has a specific role in capturing the prey. Our work on performance comparison between heterogeneous and homogeneous MAS [29] delves deeper into the problems that heterogeneity brings: our main concern was that the heterogeneous system would suffer from inferior efficiency of evolution due to the inflated search space. Moreover, the robustness of the evolved behaviour of the team of specialized predator agents would be questionable too. The reason for this is that if, for example, the team employs a dedicated “driver” agent, in real-world situations, it could be challenging to make sure that the agents would be deployed in the vicinity of the prey (i.e., a cancer cell) in such a way that the “driver” is in the most favourable position relative to the prey and other predators. Instead, we opted for an implicit behavioural heterogeneity (with genotypic homogeneity)—the agents that are the closest to the prey assume the role of the “driver”, and any of the eight predator agents may turn into this role, if needed. The heterogeneity is implicit because it arises from the interaction between the homogeneous genotype (all agents have identical four pairs of velocities of wheels) and the environment. The dynamically faced environment is what “specialises” the different predator agents in the team.

## 6. Conclusions

Nanorobots are newly emerging technology, made possible by the rapid technological advancements in the last century. Creating synthetic machines on a miniature level, however, shows that there are significant problems to overcome, due to the differences in physics laws and the limited resources available due to the small size of the robots. Furthermore, as medicine is the most prominent field of use for these new machines, they need to be reliable and precise in their work, which requires making no compromises in the quality of their operation. In attempt to solve these restrictions, we employed a variation of the predator-prey pursuit problem (PPPP), implementing very simple predator agents, equipped with a single line-of-sight sensor, and a simple control of the velocities of their two wheels. The predator agents utilize a direct mapping of the few perceived environmental states into corresponding velocities for their pair of their wheels. We applied genetic algorithms to evolve such a mapping that results in a successful capturing of the prey by the team of predator agents. However, as the preliminary results indicated, the predators featuring a straightforward sensor could not resolve more than just few of the tested initial situations. To improve the generality of the evolved behaviour, we proposed an asymmetric sensory morphology of predators, an angular offset to the sensor relative to their longitudinal axis, and coevolved both (i) the amount of this offset and (ii) the behaviour of predators. According to the experimental results, the behaviour coevolved with a sensor offset between 12° and 38° resulted in both an efficient and consistent capture of the prey in all tested initial situations. Moreover, few of the evolved behaviours for a sensor offset in the range 18°∼24° demonstrated a good generality to the variations in the speed of the prey and a good robustness to perception noise.

We believe that the obtained results could be viewed as a step towards the engineering of nanorobots with asymmetric morphology for various medical applications including pinpoint delivery of medicine, locating and destroying cancer cells, microscopic imaging, etc. In our future work, we are planning to develop a three dimensional model which will resemble a more realistic environment such as the human body.

## Figures and Tables

**Table 1:** Features of the entities used during the evolution of the behaviour of predator agents.

Feature	Predator	Prey
Number of agents	8	1
Radius (units)	8	8
Length of the axis of wheels (units)	16	16
Max linear velocity of wheels (units/s)	10	10
Max speed of agents (units/s)	10	10
Type of sensor	Single line-of-sight	Omnidirectional
Range of visibility of the sensor (units)	200	50
Orientation of sensor	Counterclockwise offset (2∼40 degrees)	—

**Table 2:** The main parameters of the GA.

Parameter	Value
Genotype	Eight integer values of the velocities of wheels (*V*_00L_, *V*_00R_, *V*_01L_, *V*_01R_, *V*_10L_, *V*_10R_, *V*_11L_, and *V*_11R_) and an integer value of the angular offset (*α*) of the sensor
Population size	400 chromosomes
Selection	Binary tournament
Selection ratio	10%
Elite	Best 4 chromosomes
Crossover	Both single- and two-point
Mutation	Single-point (with even distribution)
Mutation ratio	5%
Fitness cases	10 initial situations
Duration of the fitness trial	120 s per initial situation
Fitness value	Sum of fitness values of each situation: (a) Successful situation: time needed to capture the prey (b) Unsuccessful situation: 10,000 + the shortest distance between the prey and any predator during the trial
Termination criterion	No. of generations = 200 or stagnation of fitness for 32 consecutive generations

**Table 3:** Statistical characteristics of the 32 solutions obtained form 32 independent runs of the GA.

Parameter	Value
Mean of the best fitness values	436
Standard deviation of the best fitness value	63
Mean of the sensor offset (°)	24.7
Standard deviation of the sensor offset (°)	7.2

**Table 4:** Genotype of evolved solutions: the fastest evolved (SFE), with the best fitness (SBF), most general (SMG), most robust to FP (SMRFP), and FN (SMRFN) noise.

Solution	Fitness	*V*_00L_ (%)	*V*_00R_ (%)	*V*_01L_ (%)	*V*_01R_ (%)	*V*_10L_ (%)	*V*_10R_ (%)	*V*_11L_ (%)	*V*_11R_ (%)	Sensor offset *α* (°)
*S*_FE_ (#9)	417	30	95	100	90	−80	−75	50	−95	22
*S*_BF_ (#32)	369	−95	80	90	85	−90	−90	100	90	16
*S*_MG_ (#21)	382	−95	80	95	90	−90	−90	60	−10	24
*S*_MRFP_ (#11)	404	−70	70	90	85	−100	−100	65	70	18
*S*_MRFN_ (#14)	421	30	100	100	95	−75	−70	100	100	20

## Data Availability

The data used to support the findings of this study are included within the article.
